# Public health involvement in alcohol licensing decisions in the UK: a systematic review of qualitative studies

**DOI:** 10.1136/bmjph-2024-000953

**Published:** 2024-10-13

**Authors:** Marie Rogerson, Lindsay Blank, Mark Clowes, Emma Hock, Elizabeth Goyder

**Affiliations:** 1 Sheffield Centre for Health & Related Research (SCHARR) and School of Medicine & Population Health, The University of Sheffield, Sheffield, UK

**Keywords:** public health, public health practice, community health, preventive medicine

## Abstract

**Introduction:**

One approach to reducing alcohol consumption and related harm is to limit physical availability. However, a recent review found alcohol licensing decisions are not consistently associated with improved local health outcomes in the UK, despite public health teams (PHTs) having a statutory role in licensing. This may be explained by limitations in regulatory powers, or because PHTs have been unable to sufficiently influence the use of regulation. This review aims to synthesise qualitative evidence to understand the nature, barriers and enablers, and value of PHT involvement in alcohol licensing decisions in the UK.

**Methods:**

A systematic review of qualitative evidence was conducted. Five electronic databases were searched, supplemented by web searches for grey literature and author, reference and citation searches for included studies. Data was extracted and quality assessed using the Critical Appraisal Skills Programme (CASP) checklist. Data was synthesised using thematic synthesis, and confidence in the findings was judged using the Confidence in Evidence from Reviews of Qualitative research (CERQual) approach.

**Results:**

10 reports, relating to four separate studies, met the eligibility criteria. Thematic synthesis generated seven analytical themes. We found variation in how PHTs’ role in licensing is understood and enacted, with shared barriers and enablers. PHTs are often not regarded as a key consultee, though some teams found success in pursuing a more strategic approach. While the public health licensing objective in Scotland is considered an asset, it does not guarantee influence. Regardless of its presence, there is variation in the perceived value of PHTs’ involvement in licensing.

**Conclusions:**

A more strategic focus may be of value to PHTs and help strengthen their impact on licensing. However, given the limited potential for public health benefits through influencing regulatory decision-making, PHTs may want to consider if it is the most effective use of limited resource in tackling alcohol-related harms.

**PROSPERO registration number:**

CRD42023452508.

**WHAT IS ALREADY KNOWN ON THIS TOPIC**

**WHAT THIS STUDY ADDS**

- There are shared barriers and enablers that affect public health teams’ influence in terms of both overall strategy and individual decisions. While the public health licensing objective in Scotland is beneficial, it does not guarantee influence and there is variation in the perceived value of public health involvement in licensing.

**HOW THIS STUDY MIGHT AFFECT RESEARCH, PRACTICE OR POLICY**

- Without more effective mechanisms to influence alcohol availability, attempts to influence individual local alcohol licensing decisions are likely to have a limited impact on alcohol-related harm.

## Introduction

 Alcohol causes well-documented harms to health and well-being, exacerbating health inequalities.^1^,^3^ The risks of adverse health impacts increase with increased consumption. Limiting physical availability has been shown to reduce both consumption and its related harms.^4^ One way to do this is by licensing the sale of alcohol.^5^

In the UK, premises are required to hold a licence before they can legally sell alcohol.^6^ The system in Northern Ireland remains distinct from the rest of the UK, with a set number of licences available for granting by County Courts.^7^ In England, Scotland and Wales, local authorities (LAs) are the designated licensing authorities, with statutory responsibility for assessing applications, placing conditions on licences and developing a local Statement of Licensing Policy (SLP).^8 9^ This responsibility is delegated to the council’s licensing committee (in England and Wales) or licensing board (in Scotland).^10 11^ Licence applications can only be judged against a common set of four licensing objectives, plus an additional public health objective in Scotland.^6^

Certain bodies must be notified about all licence applications and be consulted on the SLP. They are termed responsible authorities (RAs) in England and Wales, and statutory consultees in Scotland.^12 13^ They can make representations to the licensing committee or board, and can request a licence review. Public health has been a statutory consultee since 2011, via National Health Service Health Boards, and an RA since 2013 via Local Health Boards in Wales, and Directors of Public Health, a statutory role within English LAs.^13 14^ There is no equivalent role for public health in Northern Ireland.

In Scotland, public health teams (PHTs) can seek to influence decisions for the explicit benefit of public health, in line with the corresponding licensing objective. In England and Wales, teams may attempt to influence the outcome of decisions to benefit public health, but do not have recourse to a specific objective.

Despite PHT involvement, our recent review of quantitative evidence found no consistent or sustained association between licensing decisions and local health outcomes.^15^ Limitations in current regulatory powers is one explanation for this finding, as licensing cannot, in practice, reduce the number of licensed premises.^15^ This significantly limits its potential to impact overall alcohol availability.^16^ However, it may also be that PHTs have been unable to sufficiently influence licensing decisions.

While alcohol is an important public health issue, there are a myriad of health determinants and interventions that PHTs must prioritise between. Given capacity constraints, it is important to understand the role that PHTs currently play in licensing decisions, the barriers and enablers affecting their influence and the value of PHT involvement. In addition, a qualitative exploration of how PHTs can and cannot influence licensing decisions may also help explain the limited impact of these decisions on health outcomes, as reported in our quantitative review.

This review aims to synthesise available qualitative evidence to explore the views of both PHTs and other licensing stakeholders. It seeks to answer the following question: *What are the experiences and perceptions of public health involvement in alcohol licensing decisions in the UK?* In turn, this will provide policymakers and local PHTs with evidence to inform future approaches to alcohol licensing and the strategic prioritisation of PHT resources.

## Methods

A systematic review of qualitative evidence on PHT involvement in alcohol licensing decisions in the UK was undertaken. The review was prospectively registered with PROSPERO. All stages of the review are reported in line with the Enhancing transparency in reporting the synthesis of qualitative research (ENTREQ) statement (see online supplemental material).^17^

### Eligibility criteria

The Sample, Phenomenon of Interest, Design, Evaluation, Research type (SPIDER) framework was used to define the inclusion criteria and formulate the research question.^18^ Studies were considered for inclusion if they reported on PHT involvement in alcohol licensing decisions. Experiences and perceptions of this involvement could come from PHTs themselves, or other licensing or public health stakeholders. All qualitative research methodologies and study designs (or mixed methods that included a qualitative element) were considered for inclusion. Searches were conducted for studies published from 1 January 2003 to be inclusive of any research published after legislation governing the modern alcohol licensing system in the UK was passed.^8 9^ Non-UK and non-English language evidence was excluded.

### Search strategy

The primary approach was a multi-database search of peer-reviewed journal articles. A search strategy was developed in MEDLINE using keyword searching, and adapted for EMBASE, PsycINFO, the Social Science Citation Index and the ProQuest Social Science Premium Collection. Searches were conducted in April 2023. Where available, suitable in-built filters or the validated National Institute for Health and Care Excellence (NICE) filter were used to restrict search results to UK-based studies.^19^ There are well-documented challenges in searching for qualitative studies, with a lack of validated or ‘gold standard’ filters.^20 21^ Non-qualitative studies were therefore excluded at the screening stage.

The secondary, supplementary approach sought to identify additional evidence using a range of methods. Citation searching, scrutiny of reference lists and key author searching were conducted on the reports of studies included after screening the database searches. A search of UK grey literature was also conducted, following the approach proposed by Stansfield *et al*.^22^ Scrutiny of the first 100 results from an internet search engine (Google) restricted to UK domains, combined with prior knowledge, generated a list of public health or alcohol-related organisational websites which were then searched individually. Search terms were restricted to ‘alcohol licensing’ due to limitations in search functionality.

A preliminary scoping review revealed that two of the subsequently included studies were funded by the NIHR (National Institute for Health and Care Research). The NIHR Public Health Research journal is not widely indexed by electronic databases; this journal was therefore searched separately via the NIHR journals platform.^23^

### Study selection

Electronic database search results were downloaded to a reference management system (EndNote). Duplicates were removed before screening the title and abstract of all results for eligibility against the inclusion and exclusion criteria. Where there was uncertainty about whether a paper met the inclusion criteria, a decision was made on review of the full text of the paper. Screening was carried out by one reviewer, with a 10% sample screened by a second reviewer, which confirmed the consistency of the initial sifting. For those records not excluded, full-text papers were then retrieved and screened by two reviewers. Uncertainties regarding inclusion following full-text review were resolved through discussion among the review team. Secondary searching was conducted and documented, with results similarly screened. A final list of papers was generated, grouped by the study on which they reported.

### Quality appraisal

The Critical Appraisal Skills Programme (CASP) appraisal tool for qualitative research was used to assess the validity, content and value of the included studies (see online supplemental material). The assessment was conducted by one reviewer and checked for accuracy and consistency by a second. Following Cochrane guidance, no rating or scoring system was used to summarise the assessment, and nor was the exclusion of studies considered on the basis of the assessment.^24^ Instead, the quality appraisal process was intended to enable the reviewer to engage more deeply with and better understand the methodological rigour of the selected studies, and to reflect on how any limitations may impact the review findings.^24^

### Data extraction

A standardised data extraction form was created, informed by guidance from Noyes *et al* and a NICE template for qualitative public health studies.^24 25^ The form was piloted on the first study and refined accordingly, with all data extracted and tabulated. All text under the headings ‘results’ or ‘findings’, ‘discussion’ and ‘conclusion’ was extracted, along with a range of contextual and methodological study characteristics, which are reported separately.^26^ Data was extracted by one reviewer and checked for accuracy by a second.

### Data synthesis

Thematic synthesis was used to analyse and synthesise the data, following the approach of Thomas and Harden.^27^ Coding was carried out systematically on each paper in turn, line by line, before identifying descriptive themes from the groups of codes. The third stage was an iterative, inductive process to generate the final analytical themes, which were grouped into four domains. All stages were completed consecutively by one reviewer, with the interpretation of emerging findings aided by discussion with the review team and practitioners.

The Confidence in Evidence from Reviews of Qualitative research (CERQual) approach was used to assess confidence in the review findings.^28^ In accordance with common practice, it was applied at the level of the descriptive findings, rather than the analytical themes.^29^ The four components of the assessment were completed in turn, before making an overall qualitative confidence judgement of high, moderate, low or very low.^29^

### Patient and public involvement

Consultation with practitioners and topic experts from across the UK guided the initial development of the review. It informed the focus and scope of the review question, the inclusion criteria and the identification of evidence sources. Further discussions provided insight and advice on the interpretation and implications of the findings, and dissemination to key stakeholders.

A public advisory group, consisting of seven individuals from across the UK recruited from the NIHR ‘People in Research’ website, also provided advisory input on the focus of the review, and the communication of its findings.^30^

## Results

**Figure 1:**
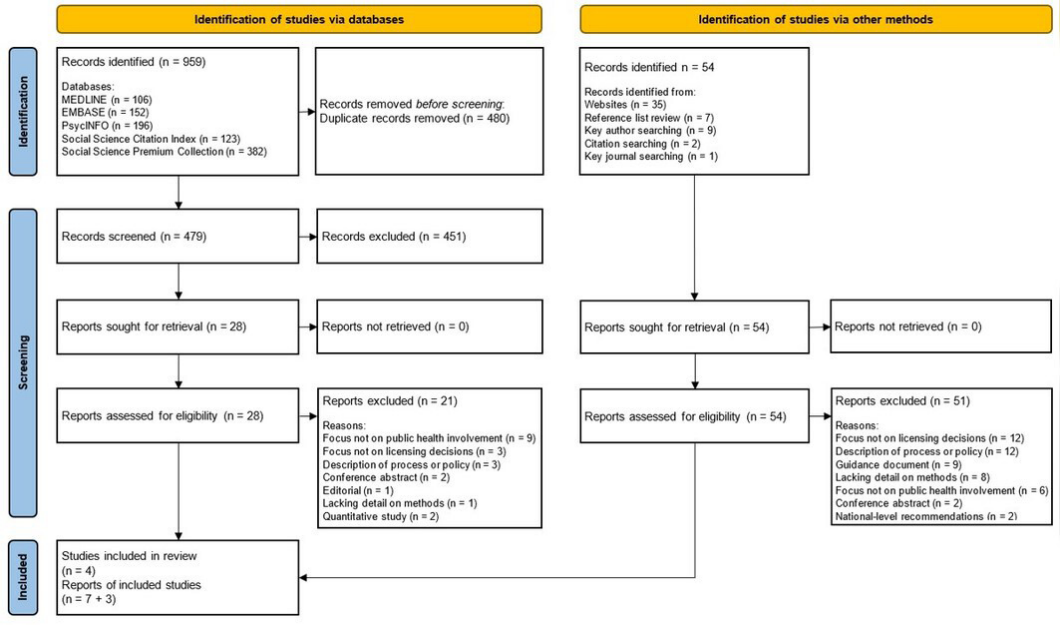
Preferred Reporting Items for Systematic review and Meta-Analysis flowchart.

Results from the searches and screening are shown in the Preferred Reporting Items for Systematic review and Meta-Analysis flowchart in figure 1.

Electronic database searches identified 959 records, of which 479 were unique. After screening, seven were selected for inclusion. A summary of the reasons for exclusion is shown in figure 1; a full list is provided in the online supplemental material. Following the selection of these seven reports, secondary searches were conducted, identifying a further 54 records. After screening, three reports were selected for inclusion. In total, 10 reports relating to four separate studies were selected for inclusion (table 1). This included eight papers published in peer-reviewed journals, one NIHR study report (2a) and one doctoral thesis (3a).

**Table 1:** Included studies

Included studies			Reports of included studies			
Title		Study lead or PI	Title		Authors	Year published
1	Qualitative interviews with public health stakeholders	PI:Fitzgerald N	1a	Democracy and power in alcohol premises licensing: A qualitative interview study of the Scottish public health objective^57^	Fitzgerald *et al*	2018
			1b	Implementing a Public Health Objective for Alcohol Premises Licensing in Scotland: A Qualitative Study of Strategies, Values, and Perceptions of Evidence^58^	Fitzgerald*et al*	2017
			1c	National objectives, local policymaking: public health efforts to translate national legislation into local policy in Scottish alcohol licensing^59^	Fitzgerald andCairney	2022
2	PHAL (Public Health and Alcohol Licensing) study	PI:Lock KStudy lead:Reynolds J	2a	Strengthening public health contributions to alcohol licensing processes. Insights from the PHAL study.^60^	Reynolds *et al*	2018
			2b	‘A true partner around the table?’ Perceptions of how to strengthen public health’s contributions to the alcohol licensing process^61^	Reynolds *et al*	2018
			2c	Processes, practices and influence: a mixed methods study of public health contributions to alcohol licensing in local government^62^	Reynolds *et al*	2018
3	Public health involvement in alcohol licensing	Study lead:Somerville L	3a	Public health involvement in alcohol licensing decisions^63^	Somerville	2018
			3b	Public health participation in alcohol licensing decisions in England: the importance of navigating “contested space”^64^	Somerville *et al*	2020
4	ExILEnS (Exploring the Impact of Licensingin England and Scotland)	PI:Fitzgerald N	4a	How public health teams navigate their different roles in alcohol premises licensing: ExILEnS multistakeholder interview findings^32^	O’Donnell *et al*	2022
			4b	‘Give us the real tools to do our jobs’: views of UK stakeholders on the role of a public health objective for alcohol licensing^65^	Nicholls *et al*	2022

PIprincipal investigator

### Study characteristics and quality appraisal

Full details of each study are provided in table 2. The four studies were conducted by different research teams, although there was a significant crossover in personnel between two studies (1 and 4), with both led by the same principal investigator. All studies were conducted between 2014 and 2020, after the point at which public health became a statutory consultee in Scotland (2011) or an RA in England (2013).

**Table 2:** Study characteristics

Study lead or PI	Year	Setting	Methods				Participants	
			Study design	Data collection	Recruitment	Data analysis	Number	Characteristics
Qualitative interviews with public health stakeholders								
PI:Fitzgerald N	2014	Scotland	Qualitative	Semi-structured interviews^(1a,1b,1c)^	Purposive and snowball sampling	Thematic analysis, inductive framework approach	N=13 (individuals)	PH practitioners from: Alcohol and drug partnerships (n=6) Local PHT within NHS (n=5) Local government licensing team (n=1) Alcohol Focus Scotland (n=1) Covered 8 of the 14 health boards in Scotland, and 20 of the 40 licensing board areas (some interviewees covered more than one area).
PHAL study								
PI:Lock KStudy lead:Reynolds J	2016–2018	London, England	Mixed methods	Ethnographic observation^(2a,2c)^	Purposive and convenience sampling	Thematic analysis, inductive coding	n=8 (PHTs in LAs)	5 inner, 3 outer LBs
				Semi-structured interviews^(2a,2c)^	Purposive and convenience sampling	Thematic analysis, inductive coding	n=10 (individuals)	3 PHT, 1 trading standards, 2 licensing, 1 police licensing officer, 1 regulatory services manager, 1 councillor, 1 barrister
				Focus groups^(2a,2b)^	Purposive and convenience sampling	Thematic analysis, inductive coding	n=37 individuals across four groups	Groups 1 & 2: 15 PHTs from 14 LBs Group 3: 8 from other RAs in 1 LB Group 4: 14 national licensing stakeholder group members
				Online survey^(2a,2b,2c)^	All 33 Greater London LAs approached	Descriptive statistics	n=18 (PHTs in LAs)	11 inner, 7 outer LBs
				Routine licensing data^(2a,2c)^	Purposive and convenience sampling	Descriptive statistics	n=4 (LAs)	5 inner LBs
Public health involvement in alcohol licensing								
Study lead:Somerville L	2015–2018	London, England	Qualitative	Semi-structured interviews^(3a,3b)^	Convenience and purposive sampling	Thematic analysis, inductive coding	n=21 (individuals)	11 PHT, 3 licensing, 2 Councillors, 1 police licensing officer, 1 regulatory services, 1 regional stakeholder, 2 national stakeholder.Covered 5 LAs and 6 LBs as the PHT in 1 LA covered 2 LBs.Covered low, medium and high engagement boroughs
				Analysis of licensing documentation^(3a,3b)^	Convenience and purposive sampling	Documentary analysis	n=8 (LAs)	Covered 11 LBs as PHTs in 3 of the LAs covered 2 LBs each
				Fieldwork notes of licensing committee meetings^(3a,3b)^	Convenience and purposive sampling	Documentary analysis	n=1 (LA)	Covered 1 LBs
ExILEnS								
PI: Fitzgerald N	2017–2020	England and Scotland	Mixed methods (*4a and 4b report only on the qualitative methods*)	Semi-structured interviews^(4a,4b)^	Purposive sampling: direct contact and initial site visits, and snowball sampling	Thematic analysis using deductive and inductive coding	n=53 (individuals)	28 PHT^(4a,4b)^25 licensing^(4a)^Covers 20 LAs, 14 in England, 6 in Scotland

Labels 1a, 1b etc, refer to the reports of studies listed in table 1Synthesis findings.

ExILEnSExploring the Impact of Licensing in England and ScotlandLA, local authority; LBLondon boroughPHALPublic Health and Alcohol LicensingPHT, public health team; PI, principal investigator

There is a strong degree of commonality between the core research aims across the four studies. Two studies (1 and 3) employed a qualitative design, while two (2 and 4) were mixed methods. Recruitment methods were similar across the studies and all four used semi-structured interviews. In addition, one study (2) used focus groups and ethnographic observation, and another (3) carried out documentary analysis on fieldwork notes and licensing documentation.

Two studies (2 and 3) were set in LAs in London, one study (1) was set solely in Scotland, and the final study (4) covered both England and Scotland. All four studies sought individuals working in PHTs who, at a minimum, had the potential to be involved in local alcohol licensing decisions. One study (3) actively sought to recruit individuals from a mix of low, medium and high engagement areas. In addition to public health actors, three studies (2–4) recruited practitioners from licensing and/or other RAs.

Full results of the CASP quality appraisal are presented in the online supplemental material. No significant methodological limitations in the studies were found.

### Synthesis findings

**Figure 2:**
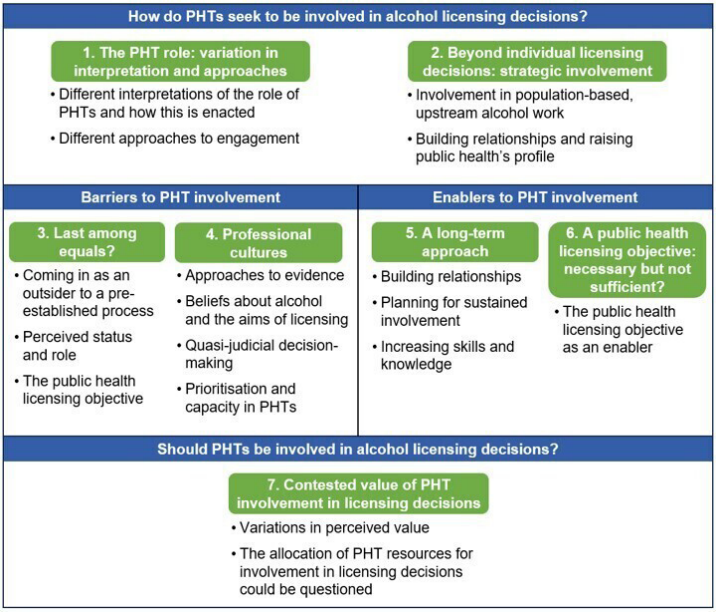
Analytical and descriptive themes by domain. PHT, public health team.

The thematic synthesis identified 17 descriptive findings, grouped into four domains, from which seven analytical themes were generated (figure 2). These seven themes are the central concepts that comprise the findings and the substance of the analytical narrative. However, as recognised in methods literature, an overarching level of demarcation can provide additional structure, context and clarity.^31^ During the synthesis it became clear that there was organisational and interpretative value in grouping the seven themes into four domains, exploring the methods, barriers and enablers, and evaluative dimensions of PHT involvement in licensing, and these are used later to structure our discussion.

Summaries of the descriptive findings are provided in table 3, alongside the results of the CERQual assessment of these findings. Confidence was generally high (in 13 out of 17), with moderate confidence in the others (4 out of 17). Commentary on the four components contributing to these judgements is provided in the full CERQual evidence profile (see online supplemental material).

**Table 3:** CERQual summary of qualitative findings

Summary of review finding	Reports of studies contributing to the review finding	CERQual assessment of confidence in the evidence	Explanation of CERQual assessment
The PHT role: variation in interpretation and approaches			
There are different interpretations of the role of PHTs and the way teams enact this through the work they do.	1a,b; 2a,c; 3a,b; 4a	High confidence	Three studies with minor concerns regarding methodological limitations. No or very minor concerns in the other domains.
PHTs demonstrate differing approaches to engagement with other licensing stakeholders.	1b; 2a; 3a,b; 4a	High confidence	Three studies with minor concerns regarding methodological limitations. No or very minor concerns in the other domains.
Beyond individual licensing decisions: strategic involvement			
Some PHTs prioritise building relationships and raising public health’s profile over trying to influence individual licensing decisions.	1b; 2a,c; 3a; 4a,b	High confidence	Four studies with minor concerns regarding methodological limitations and coherence, but no or very minor concerns in the other domains.
Some PHTs prioritise involvement in upstream, population-based work such as SLPs and CIPs, rather than individual licensing decisions.	2a,b,c; 4a,b	High confidence	Two studies with minor concerns regarding methodological limitations and adequacy, but no or very minor concerns in the other domains.
Last among equals?			
PHTs have faced barriers as a result of coming in as an outsider to licensing, which has pre-established processes and actors.	1a,c; 3a,b	Moderate confidence	Two studies, with moderate concerns regarding adequacy and minor concerns regarding methodological limitations. No or very minor concerns in the other domains.
PHTs are perceived as having an unequal status and a more supporting role compared with other licensing stakeholders.	1a,c; 2a,b; 3a,b; 4a,b	High confidence	Four studies with minor concerns regarding methodological limitations and coherence, but no or very minor concerns in the other domains.
The public health licensing objective is perceived, in Scotland, to be a lesser priority; in England, its absence means public health considerations appear less valued.	1b,c; 2a; 3a,b; 4a, 4b	High confidence	Four studies with minor concerns regarding methodological limitations and coherence. No or very minor concerns in the other domains.
Professional cultures			
There are differences in approaches between PHTs and other licensing stakeholders as to how evidence is defined and what is considered valuable and relevant.	1b,c; 2a,b; 3a,b; 4a	High confidence	Four studies with no or very minor concerns across the four domains.
There are differences in beliefs about alcohol and the aims of licensing between PHTs and other licensing stakeholders.	1b,c; 2a; 3a; 4a	High confidence	Four studies with minor concerns regarding coherence. No or very minor concerns across the other domains.
The licensing process is a form of quasi-judicial decision-making, and is unfamiliar to PHTs.	1a,c; 2a; 3a	High confidence	Three studies, with minor concerns regarding coherence, and minor concerns regarding methodological limitations in two. No or very minor concerns in the other domains.
The prioritisation of licensing work and the capacity within PHTs to undertake it.	1c; 2a,b,c	Moderate confidence	Two studies with moderate concerns regarding adequacy and methodological limitations. No or very minor concerns in the other domains.
A long-term approach			
Taking the time to build relationships with other licensing stakeholders is seen as beneficial for PHTs.	1b,c; 2a,b; 3a,b; 4a,b	High confidence	Four studies with no or very minor concerns across the four domains.
It is beneficial for PHTs to plan for licensing work to require sustained involvement.	1c; 2a; 3a,b	Moderate confidence	Three studies with moderate concerns regarding coherence and adequacy. No or very minor concerns regarding relevance or methodological limitations.
It is beneficial for PHTs to increase their relevant knowledge and skills, such as understanding of the licensing process and communication of evidence.	1c; 2a,b; 3a,b	High confidence	Three studies with minor concerns regarding adequacy. No or very minor concerns in the other three domains.
A public health licensing objective: necessary but not sufficient?			
A public health licensing objective is seen as beneficial, if not necessary, in theory in England and in practice in Scotland, but it is not seen as sufficient for effective PH involvement.	1a,b,c; 2a,b; 3a,b; 4b	High confidence	Four studies with minor concerns regarding methodological limitations and coherence. No or very minor concerns regarding adequacy and relevance.
Contested value of PHT involvement			
There are variations among PHTs and other licensing stakeholders in the perceived value of PHT involvement in licensing decisions.	1a,b; 2a,b,c; 3a; 4a	High confidence	Four studies with minor concerns in three regarding methodological limitations and in one regarding adequacy, but these concerns do not threaten the finding itself as variation is demonstrated regardless. No or very minor concerns regarding coherence or relevance.
The allocation of PHT resources for involvement in licensing decisions could be questioned in light of PHTs’ potentially limited impact.	1b, 2c; 3a; 4a	Moderate confidence	Four studies with moderate concerns regarding coherence, adequacy and methodological limitations. No or very minor concerns regarding relevance.

CERQualConfidence in Evidence from Reviews of Qualitative researchCIP, cumulative impact policies; PHT, public health team; SLP, Statement of Licensing Policy

The analytical themes are expanded on below, with selected quotations provided in support.

### Theme 1: the PHT role: variation in interpretation and approaches

Studies found there were differing interpretations of PHTs’ role, among both PHTs themselves, and other licensing stakeholders. There was a split between those who thought PHTs should play a primarily supportive role, and those who thought it should be proactively and independently seeking to influence decisions to reduce alcohol availability. A third approach prioritised collaboration with other licensing stakeholders. These differences in interpretation were also visible in how PHTs enacted their role, through the work and engagement they pursued. One study described this as a ‘tension’ as to whether PHTs’ activity should be:

[…] aimed to help the licensing board understand local data—as a kind of neutral support, providing ‘impartial’ advice—or used [to] actively to influence them towards decisions felt to favour public health. 1b, author

In Scotland, the existence of the public health licensing objective adds an extra dimension to PHTs’ approaches and rationale, with one study (4) finding that all Scottish participants had tried, to some extent, to take a ‘challenging’ approach. However, this has not resulted in uniformity. Some teams were selective, whereas others felt obligated to respond to every application.

### Theme 2: beyond individual licensing decisions: strategic involvement

Studies found many PHTs took a more strategic approach to alcohol licensing work, sometimes at the expense of individual licensing applications. For some teams, this entailed prioritising building relationships with other licensing stakeholders and, in England, using licensing work to raise the profile of public health within the LA. Teams in both England and Scotland have reported success in focusing more on upstream, population-based licensing work, such as SLPs and cumulative impact policies (CIPs):

We have influenced the policy and influenced where the cumulative impact zones are […]. I think that’s where we added, you know, we made the most difference. 2c, participant

### Theme 3: last among equals?

One set of barriers to PHT involvement identified from the studies conveyed a strong sense of PHTs being ‘last among equals’. PHTs came in as an outsider to the licensing system, which had pre-established processes and actors, and was therefore difficult to enter and influence. Despite its now equal statutory role, PHTs are perceived to have a lower status than other licensing stakeholders:

Theoretically we’ve got the same say as every other Responsible Authority. But it doesn’t feel like that still. 3a, participant

This was particularly true in England, where the lack of a licensing objective is seen by both PHTs and other licensing stakeholders to contribute significantly to their lower status. In Scotland, the licensing objective is seen as giving PHTs more credibility, although it has not led to public health concerns carrying equal weight when influencing licensing decisions.

### Theme 4: professional cultures

Further barriers related to the impact of professional cultures within licensing and public health. There are clear differences in how evidence is defined, and what is considered valuable and relevant. Public health typically takes a population, data-driven perspective, whereas the licensing legislation requires a focus on individual premises, leading one participant to conclude:

it’s personal stories and testimony that the licensing sub-committee pay attention to not to data and statistics. 3b, participant

There are also fundamental differences in beliefs about alcohol, public health and the aims of licensing, with an evident tension between prioritising economic growth and reducing alcohol-related harms. In addition, licensing is a form of quasi-judicial decision-making, with formal, legalistic cultures and conventions that are unfamiliar to PHTs. The usual influence that public health professionals have is diminished, with power residing among licensing clerks, legal specialists and businesses with the financial resources to threaten costly legal challenges. Finally, licensing is not always a priority for PHTs, and they have limited capacity to take on new work.

### Theme 5: a long-term approach

The studies identified a number of enablers to PHT involvement, many of which were connected with the notion of taking a long-term approach.

[…] there’s a hearts and minds element of it and part of that is about the passage of time. 1c, participant

One aspect was recognising the need for sustained involvement. This requires dedicating sufficient resources, having a senior ‘champion’ and actively retaining institutional knowledge of licensing when PHT members change. The second aspect was taking the time to build relationships with other licensing stakeholders, and recognising that this an important aspect of influencing. Third, it was considered beneficial for PHTs to take the time to build their knowledge and skills around licensing.

### Theme 6: a public health licensing objective: necessary but not sufficient?

The role of a public health licensing objective as an enabler, in theory in England and in practice in Scotland, emerged from the studies as a distinctive theme. It was considered advantageous in Scotland for both licensing decisions and more strategic work:

Getting people to start thinking, or boards to start thinking, a bit wider than just the case by case. […] having that objective in there has really been supportive for us. 4b, participant

It was not considered necessary by all English PHTs, but many thought an objective would be beneficial in increasing PHTs’ profile and influence within licensing. However, despite its perceived benefits, a licensing objective was not seen as sufficient for effective PHT involvement. This supports findings from Scotland, where the presence of a public health licensing objective has not guaranteed effective involvement or resulted in a transformation within licensing decision-making.

### Theme 7: contested value of PHT involvement in licensing decisions

The studies demonstrated that there are variations among PHTs and other licensing stakeholders as to the perceived value of PHT involvement in licensing decisions. While some felt they had had a direct, positive impact, many others were more sceptical, ‘often reflecting on it in fairly negative terms’ (2a). This resulted in all studies at least raising the issue of whether the allocation of PHT resources for involvement in licensing decisions should be questioned in light of PHTs’ potentially limited impact:

There were mixed views, both amongst interviewees, and in their reports of the views of others, on whether licensing could actually make a difference to public health. For these reasons one participant questioned whether all the effort on overprovision was ‘worth the time invested’. 1b, author

## Discussion

This is the first qualitative review of PHT involvement in alcohol licensing in the UK. We found variation in how the role of PHTs in licensing is understood and enacted, with some prioritising more strategic involvement. We identified sets of barriers and enablers to PHT involvement, and found variation in the perceived value of this, suggesting the allocation of PHT resources to licensing work could be justifiably questioned.

### Current PHT involvement

There is a lack of consensus regarding PHTs’ role within licensing, particularly in England. Studies were skewed towards higher activity areas, inevitably under-emphasising teams who have minimal to no involvement. In areas where there is engagement, many teams and licensing stakeholders expect PHTs to play a supportive role, centred on providing data. Others perceive a more proactive role, which might entail: meeting regularly with licensing stakeholders; consulting with residents; reviewing applications; negotiating with applicants; and submitting representations. Challenging applications are more common in Scotland, though, over time, Scottish teams have shifted to more collaborative working with other licensing stakeholders.^32^

This is one aspect of teams taking a more strategic approach, where relationships are developed over time and sometimes prioritised at the expense individual representations. The other aspect is influencing local licensing policy development. The potential for PHTs to influence SLPs, CIPs and overprovision assessments was recognised by early policy reviews in England and Scotland.^33^,^35^ The findings from this synthesis demonstrate that some teams are indeed pursuing this approach, though separate quantitative analysis from the ExILEnS (Exploring the Impact of Licensing in England and Scotland) study (4) suggests this is more common in Scotland.^36^ Other temporal trends have been observed from this analysis, with activity levels increasing over time in both lower and higher activity areas, but remaining higher in Scotland than England throughout.^36^

### Barriers and enablers

Numerous barriers to involvement were identified, linked to both differing professional cultures, and the processes and perceptions that render PHTs a junior partner in the licensing system, despite their equal statutory role. These findings are echoed by reports from government and non-governmental sources assessing the local implementation of alcohol licensing legislation in England and Scotland.^33 34 37 38^ While these barriers have been widely reported, established policy theory suggests they should perhaps have been anticipated. As Nicholls contends, alcohol licensing is a form of multilevel governance, where power has been dispersed from national to local government and other local stakeholders.^33^ He goes on to suggest that licensing is moreover an example of Lipsky’s ‘street level bureaucracy’,^39^ where local officials exercise a notable degree of discretion in how they apply the law.^33^ This inevitably leads to an unequal distribution of power, with local networks and cultures emerging in response.^33^ Even though partnership working is at the core of public health practice, it is understandable that teams have faced barriers trying to influence this policy environment as outsiders to established networks, who may not know the unwritten rules of engagement.^40 41^

The quasi-legal nature of the system compounds these challenges, as different types of evidence, skills and resources are valued compared with those prioritised in public health. Public health traditionally promotes evidence-based policymaking, but political scientists have criticised the naivety of this approach for assuming rational decision-making, and that the definition or merit of evidence is uncontested.^33^ Indeed, as this review has found, epidemiological data, the cornerstone of public health practice, is not generally considered important in a licensing context. Work undertaken by Public Health England to pilot and evaluate a data tool did provide PHTs with more easily accessible, granular health information, but this did not overcome the preference for premise-level cause and effect data, narratives or testimonies within licensing hearings.

The legalistic environment is also comparatively favourable to business and the alcohol industry. Parallels can be drawn with the policy dystopia model created to explain tobacco industry influence, which shows how the industry creates a narrative of policy failure and adverse social and economic consequences, disseminated through multiple methods to maximise influence over policy decisions.^42^ Within licensing, businesses use both of these discursive and instrumental tactics to influence decision-makers. They promote a narrative that emphasises economic considerations, reinforcing concerns of locally elected members, while access to legal expertise and the threat of litigation to appeal a decision puts obvious pressure on decision-makers. Though small businesses may not have access to such resources, there are examples of the alcohol industry leveraging its power to challenge local decisions, such as the attempts to introduce Early Morning Restriction Orders in various English LAs.^33^ Fear of legal challenge can deter similar policies or decisions in other localities, illustrating the pervasive power of commercial determinants on health.

The role of a public health licensing objective to help balance this competing agenda remains unclear. In line with previous policy analysis and surveys, this review found the absence of a licensing objective is seen as a substantial barrier in England, and that there is demand from public health for one to be introduced.^34 38 43 44^ However, expectations of how transformational such an objective would be are moderated by the Scottish experience: though the objective is valued by PHTs, its application to individual decisions has not been straightforward.^34 36 37^ The presence of a licensing objective is assumed to account for the higher levels in Scotland of PHT activity and engagement in challenging applications. This suggests that, while not a panacea, it does promote greater involvement.

Other enablers identified by this review, centred on taking a long-term approach, are well supported by the literature and the policy theory outlined above, including the mock hearings conducted during the pilot of Public Health England’s local data tool.^38^ ^44^ Taking the time to gain knowledge of the system, understand how best to communicate and engage, and build relationships is considered fundamental for influencing in any political sphere.^40^ However, as in the case of alcohol licensing, this is especially true when multiple stakeholders are competing for influence over officials who are empowered to use their discretion in how policy is enacted locally.

### Should PHTs be involved?

Despite the passage of time, this review found that variations remain in the perceived value of PHT involvement in licensing. Some stakeholders see merit, particularly in upstream policies such as SLPs and in raising public health’s profile, and some teams have had success in influencing individual decisions. Conversely, others see little benefit, and question whether meaningful influence is practicable. This conclusion is understandable given the current legal framework and fundamental differences between traditional licensing and public health perspectives. Public health proponents advocate for a population health approach, which recognises alcohol-associated harms to health and well-being. They look to licensing as a means of reducing availability and thereby consumption. In contrast, a conventional licensing perspective considers applications on a case-by-case basis, guided by the legal framework. Even with a public health licensing objective, which allows applications to be judged against the protection or promotion of public health, there remains, in practice, a conflict with current licensing requirements. Ultimately, licensing applications are judged on the basis of a specific premise and its potential impact, requiring the cause and effect of any related harms to be demonstrated at a premise, rather than epidemiological level. This makes it extremely difficult to take public health considerations into account, as such data is unattainable. Indeed, this was the justification for not recommending such an objective be introduced when English licensing legislation was reviewed by a House of Lords Select Committee in 2017.^45^

Even with more strategic interventions, such as public health-informed SLPs and CIPs, under the current regulatory framework this could, at most, maintain current levels of licensed premises. There are restricted conditions under which licensing boards or committees can review existing licences; their primary power rests on rejecting new applications, and does not address online sales.^16^ The potential impact on overall alcohol availability is therefore extremely limited. Given this, it is unclear whether licensing could ever meaningfully reduce consumption. As the findings from our recent quantitative review suggest, licensing may not be the most effective mechanism for reducing population harms.^15^

This ultimately leads to the question of whether PHTs should be prioritising the allocation of resources for influencing licensing. Licensing is not the only approach to reducing alcohol harm, and alcohol is but one of many health determinants. In the context of competing priorities, diminished public health budgets and the time commitment required to engage successfully, this suggests that involvement in local licensing processes may not be a worthwhile use of limited PHT resources. Instead, as we discuss below, national policy mechanisms should be pursued, supported by public health professionals working at that level.^46^

### Strengths and limitations

This is the first qualitative review of PHT involvement in licensing decisions in the UK, exploring perspectives from both PHTs and other licensing stakeholders. Restricting the review to UK evidence enabled a detailed exploration of PHT involvement in a similar regulatory system (excluding Northern Ireland), where all had a statutory role. It also enabled and a comparison between PHTs who had recourse to a public health licensing objective, and those who did not.

Restricting the review to UK evidence was also a limitation. Only a small number of studies were suitable for inclusion. One was set in Scotland, two in England, and one in both Scotland and England. However, the two England-only studies were both set in London, giving a potentially unrepresentative picture of the rest of the country. There were no studies from Wales or Northern Ireland. Collectively, this means that any UK-wide generalisations, or comparisons between countries, must be treated with some caution. Similarly, the findings cannot be generalised to countries with different regulatory systems.

Studies were also skewed towards areas where PHTs have been more involved in licensing. Only one sought to recruit a mix of high, medium and low engagement areas; two intentionally excluded those with no or minimal engagement, and the third suggested their recruitment was likely to be biased towards more engaged areas. This affected the confidence in certain findings, particularly regarding barriers to involvement. Reasons why a team may not be engaged in licensing work can be inferred from the findings, but the small sample size of participants from low engagement areas means generalisations cannot be made with confidence.

### Implications for policy, practice and future research

Findings from this review suggest there may be some strategic value for PHTs in building relationships with licensing stakeholders and promoting consideration of public health, particularly through SLPs. However, in light of the limited impact of licensing on population health and competing public health priorities, involvement in individual licensing decisions may not be an effective use of resources. Teams may therefore wish to consider reviewing their local strategy for reducing alcohol-related harms.

Similarly, it is not clear that a public health licensing objective has or would have a significant impact on population health, given the current requirement of licensing decisions to be based on an individual premise and its potential impact. There are also trade-offs in advocating for legislative changes in this area, when there are competing public health priorities that would benefit from parliamentary attention.^47^ Lobbying for the introduction of a public health objective in England and Wales is therefore unlikely to be an effective use of PHT resources and political capital.

International evidence suggests other regulatory approaches to limiting physical availability, particularly through reduced hours of sales, can be effective, although more restrictive policies can lead to unintended consequences.^48^,^50^ Evaluations of the impact of the Licensing Act 2003, which deregulated trading hours in England and Wales, have been mixed, and opening hours have recently been extended in two Scottish cities.^51^ It is therefore not considered a policy priority by the UK’s Alcohol Health Alliance.^52^

Other national policy mechanisms for reducing alcohol harms should, however, be pursued, and advocated for by public health professionals operating at a national level.^52^ Evidence suggests policies that reduce affordability are the most effective and cost-effective measures.^4 48^ The introduction of a minimum unit price for alcohol in Scotland has been associated with a reduction in harms, but only Wales has replicated this.^53^ Similarly, it is estimated that reinstating the alcohol duty escalator would bring significant population health benefits.^54^ A complementary strategy would be to adopt a comprehensive approach to tackling the commercial determinants of health, guided by recent recommendations.^55 56^

Without significant regulatory change, further research on PHT involvement in licensing decisions in the UK is unlikely to identify significant opportunities for greater impact.

## Conclusions

There is variation in how PHTs’ role in licensing is understood and enacted in England and Scotland, with shared barriers and enablers to PHT involvement in both countries. PHTs are often not regarded as a key consultee, still perceived as an outsider, though some teams have found success in taking a more strategic approach. The presence of a public health licensing objective in Scotland is a distinguishing feature, but although it is considered to be an asset, it does not guarantee influence. Regardless of its presence, there is variation in the perceived value of PHT involvement in licensing. Having a more strategic focus may offer value to PHTs and help strengthen their impact, but requires a substantial time commitment.

Ultimately, the ability of licensing to meaningfully reduce alcohol availability and population harms is seriously constrained, even with a public health licensing objective and an informed influencing strategy. Given the limited potential for public health benefit, PHTs may want to consider if influencing regulatory decision-making is the most effective use of limited resources in tackling alcohol-related harms.

## supplementary material

10.1136/bmjph-2024-000953 online supplemental file 1

## Data Availability

Data sharing not applicable as no data sets generated and/or analysed for this study. All data relevant to the study are included in the article or uploaded as supplementary information.
