# Supplementary material for: Public health involvement in alcohol licensing decisions in the UK: a systematic review of qualitative studies
Source: BMJ Public Health. 2024 Oct 13;2(2):e000953. doi: 10.1136/bmjph-2024-000953 (PMC11816881; doi:10.1136/bmjph-2024-000953)
Supplement: online supplemental file 1 [file bmjph-2-2-s001.pdf]

Supplementary material

1. **ENTERQ statement**
2. **Example search strategy**
3. **Electronic sources searched**
4. **List of excluded papers and reasons for exclusion**
5. **Quality appraisal results**
6. **CERQual Qualitative Evidence Profile**

1. ENTREQ statement

	**Item**	**Guide and description**	**Location in review**
**1**	**Aim**	State the research question the synthesis addresses.	Background
**2**	**Synthesis methodology**	Identify the synthesis methodology or theoretical framework which underpins the synthesis, and describe the rationale for choice of methodology *(e.g. meta-ethnography, thematic synthesis, critical interpretive synthesis, grounded theory synthesis, realist synthesis, meta-aggregation, meta-study, framework synthesis).*	Methods
**3**	**Approach to searching**	Indicate whether the search was pre-planned (*comprehensive search strategies to seek all available studies)* or iterative (*to seek all available concepts until they theoretical saturation is achieved)*.	Methods
**4**	**Inclusion criteria**	Specify the inclusion/exclusion criteria *(e.g. in terms of population, language, year limits, type of publication, study type).*	Methods
**5**	**Data sources**	Describe the information sources used (e.g. *electronic databases (MEDLINE, EMBASE, CINAHL, psycINFO, Econlit), grey literature databases (digital thesis, policy reports), relevant organisational websites, experts, information specialists, generic web searches (Google Scholar) hand searching, reference lists)* and when the searches conducted; provide the rationale for using the data sources.	Methods Supplementary material
**6**	**Electronic Search strategy**	Describe the literature search *(e.g. provide electronic search strategies with population terms, clinical or health topic terms, experiential or social phenomena related terms, filters for qualitative research, and search limits)*.	Methods Supplementary material
**7**	**Study screening methods**	Describe the process of study screening and sifting *(e.g. title, abstract and full text review, number of independent reviewers who screened studies).*	Methods
**8**	**Study characteristics**	Present the characteristics of the included studies *(e.g. year of publication, country, population, number of participants, data collection, methodology, analysis, research questions).*	Results Table 2

**9**	**Study selection results**	Identify the number of studies screened and provide reasons for study exclusion *(e,g, for comprehensive searching, provide numbers of studies screened and reasons for exclusion indicated in a figure/flowchart; for iterative searching describe reasons for study exclusion and inclusion based on modifications t the research question and/or contribution to theory development).*	Results<br>Figure 1<br>Supplementary material
**10**	**Rationale for appraisal**	Describe the rationale and approach used to appraise the included studies or selected findings *(e.g. assessment of conduct (validity and robustness), assessment of reporting (transparency), assessment of content and utility of the findings).*	Methods
**11**	**Appraisal items**	State the tools, frameworks and criteria used to appraise the studies or selected findings *(e.g. Existing tools: CASP, QARI, COREQ, Mays and Pope* [25]*; reviewer developed tools; describe the domains assessed: research team, study design, data analysis and interpretations, reporting).*	Methods
**12**	**Appraisal process**	Indicate whether the appraisal was conducted independently by more than one reviewer and if consensus was required.	Methods
**13**	**Appraisal results**	Present results of the quality assessment and indicate which articles, if any, were weighted/excluded based on the assessment and give the rationale.	Results<br>Supplementary material
**14**	**Data extraction**	Indicate which sections of the primary studies were analysed and how were the data extracted from the primary studies? *(e.g. all text under the headings "results /conclusions" were extracted electronically and entered into a computer software).*	Methods
**15**	**Software**	State the computer software used, if any.	Methods
**16**	**Number of reviewers**	Identify who was involved in coding and analysis.	Methods
**17**	**Coding**	Describe the process for coding of data *(e.g. line by line coding to search for concepts).*	Methods
**18**	**Study comparison**	Describe how were comparisons made within and across studies *(e.g. subsequent studies were coded into pre-existing concepts, and new concepts were created when deemed necessary).*	Methods
**19**	**Derivation of themes**	Explain whether the process of deriving the themes or constructs was inductive or deductive.	Methods
**20**	**Quotations**	Provide quotations from the primary studies to illustrate themes/constructs, and identify whether the quotations were participant quotations of the author's interpretation.	Results
**21**	**Synthesis output**	Present rich, compelling and useful results that go beyond a summary of the primary studies (e.g. *new interpretation, models of evidence, conceptual models, analytical framework, development of a new theory or construct).*	Results<br>Figure 2<br>Discussion

2. Example search strategy

Ovid MEDLINE(R) ALL 1946 to April 24, 2023

1. (alcohol adj3 (licen* or decision*)).mp.
2. alcohol.mp.
3. (licen* adj2 (decision* or polic*)).mp.
4. (local adj2 alcohol polic*).mp.
5. ("cumulative impact polic*" or "cumulative impact zone*" or "cumulative impact area*").mp.
6. exp United Kingdom/
7. (national health service* or nhs*).ti,ab,in.
8. (english not ((published or publication* or translat* or written or language* or speak* or literature or citation*) adj5 english)).ti,ab.
9. (gb or "g.b." or britain* or (british* not "british columbia") or uk or "u.k." or united kingdom* or (england* not "new england") or northern ireland* or northern irish* or scotland* or scottish* or ((wales or "south wales") not "new south wales") or welsh*).ti,ab,jw,in.
10. (bath or "bath's" or ((birmingham not alabama*) or ("birmingham's" not alabama*) or bradford or "bradford's" or brighton or "brighton's" or bristol or "bristol's" or carlisle* or "carlisle's" or (cambridge not (massachusetts* or boston* or harvard*)) or ("cambridge's" not (massachusetts* or boston* or harvard*)) or (canterbury not zealand*) or ("canterbury's" not zealand*) or chelmsford or "chelmsford's" or chester or "chester's" or chichester or "chichester's" or coventry or "coventry's" or derby or "derby's" or (durham not (carolina* or nc)) or ("durham's" not (carolina* or nc)) or ely or "ely's" or exeter or "exeter's" or gloucester or "gloucester's" or hereford or "hereford's" or hull or "hull's" or lancaster or "lancaster's" or leeds* or leicester or "leicester's" or (lincoln not nebraska*) or ("lincoln's" not nebraska*) or (liverpool not (new south wales* or nsw)) or ("liverpool's" not (new south wales* or nsw)) or ((london not (ontario* or ont or toronto*)) or ("london's" not (ontario* or ont or toronto*)) or manchester or "manchester's" or (newcastle not (new south wales* or nsw)) or ("newcastle's" not (new south wales* or nsw)) or norwich or "norwich's" or nottingham or "nottingham's" or oxford or "oxford's" or peterborough or "peterborough's" or plymouth or "plymouth's" or portsmouth or "portsmouth's" or preston or "preston's" or ripon or "ripon's" or salford or "salford's" or salisbury or "salisbury's" or sheffield or "sheffield's" or southampton or "southampton's" or st albans or stoke or "stoke's" or sunderland or "sunderland's" or truro or "truro's" or wakefield or "wakefield's" or wells or westminster or "westminster's" or winchester or "winchester's" or wolverhampton or "wolverhampton's" or (worcester not (massachusetts* or boston* or harvard*)) or ("worcester's" not (massachusetts* or boston* or harvard*)) or (york not ("new york*" or ny or ontario* or ont or toronto*)) or ("york's" not ("new york*" or ny or ontario* or ont or toronto*))))).ti,ab,in.

11. (bangor or "bangor's" or cardiff or "cardiff's" or newport or "newport's" or st asaph or "st asaph's" or st davids or swansea or "swansea's").ti,ab,in.

12. (aberdeen or "aberdeen's" or dundee or "dundee's" or edinburgh or "edinburgh's" or glasgow or "glasgow's" or inverness or (perth not australia*) or ("perth's" not australia*) or stirling or "stirling's").ti,ab,in.

13. (armagh or "armagh's" or belfast or "belfast's" or lisburn or "lisburn's" or londonderry or "londonderry's" or derry or "derry's" or newry or "newry's").ti,ab,in.

14. or/6-13

15. (exp africa/ or exp americas/ or exp antarctic regions/ or exp arctic regions/ or exp asia/ or exp australia/ or exp oceania/) not (exp United Kingdom/ or europe/)

16. 14 not 15

17. 1 or (2 and 3) or 4 or (2 and 5)

18. 16 and 17

19. limit 18 to yr="2003 -Current"

3. Electronic sources searched

Electronic databases (search platform)	Organisational websites	Key journals
EMBASE (OVID)<br>MEDLINE (OVID)<br>Social Science Citation Index (Web of Science)<br>PsycINFO (OVID)<br>Social Science Premium Collection (ProQuest)	Alcohol Change UK<br>Alcohol Focus Scotland<br>Alcohol Health Alliance<br>Association of the Directors of Public Health<br>Alcohol Licensing Community<br>Faculty of Public Health<br>Office for Health Improvement and Disparities<br>Institute of Alcohol Studies<br>Local Government Association<br>Medical Council on Alcohol<br>NIHR School for Public Health Research<br>Public Health Scotland<br>Public Health Wales<br>Public Health Agency (Northern Ireland)<br>Royal Society for Public Health<br>Scottish Health Action on Alcohol Problems	Public Health Research (NIHR Journals Library)

4. List of excluded papers and reasons for exclusion

Electronic database searching

Report of study	Reason for exclusion
David G, Cooper R, Dixon S, Holmes J. Exploring the implementation of public involvement in local alcohol availability policy: the case of alcohol licensing decision-making in England. ADDICTION. 2022;117(4):1163-72.	Not focused on public health involvement
Egan M, Brennan A, Buykx P, De Vocht F, Gavens L, Grace D, et al. Local policies to tackle a national problem: Comparative qualitative case studies of an English local authority alcohol availability intervention. HEALTH & PLACE. 2016;41:11-8.	Not focused on public health involvement
Fitzgerald N, Mohan A, Maani N, Purves R, de Vocht F, Angus C, et al. Measuring how PH stakeholders seek to influence alcohol premises licensing in England and Scotland: the Public Health engagement In Alcohol Licensing (PHIAL) measure. Journal of studies on alcohol and drugs. 2022.	Quantitative
Gavens L, Holmes J, Buykx P, de Vocht F, Egan M, Grace D, et al. Processes of local alcohol policy-making in England: Does the theory of policy transfer provide useful insights into public health decision-making? Health & Place. 2019;57:358.	Description of process or policy (policy transfer)
Grace D, Egan M, Lock K. Examining local processes when applying a cumulative impact policy to address harms of alcohol outlet density. HEALTH & PLACE. 2016;40:76-82.	Not focused on public health involvement
Herring R, Thom B, Foster J, Franey C, Salazar C. Local responses to the Alcohol Licensing Act 2003: The case of Greater London. Drugs: Education, Prevention and Policy. 2008;15(3):251-65.	Not focused on public health involvement
Humphreys DK, Smith DM. Alcohol licensing data: Why is it an underused resource in public health? Health & Place. 2013;24:110-4.	Not focused on public health involvement
Lesch M, McCambridge J. Reconceptualising the study of alcohol policy decision-making: the contribution of political science. ADDICTION RESEARCH & THEORY. 2021;29(5):427-35.	Not focused on licensing decisions
Martineau FP, Graff H, Mitchell C, Lock K. Responsibility without legal authority? Tackling alcohol-related health harms through licensing and planning policy in local government. JOURNAL OF PUBLIC HEALTH. 2014;36(3):435-42.	Description of process or policy (legal and policy framework)
McGrath M, Reynolds J, Smolar M, Hare S, Ogden M, Popay J, et al. Identifying opportunities for engaging the 'community' in local alcohol decision-making: A literature review and synthesis. The International Journal on Drug Policy. 2019;74:193.	Not focused on public health involvement
Mistral W, Velleman R, Templeton L, Mastache C. Local Action to Prevent Alcohol Problems: Is the UK Community Alcohol Prevention Programme the Best Solution? International Journal of Drug Policy. 2006;17(4):278-84.	Not focused on licensing decisions
Mooney JD, Sattar Z, De Vocht F, Smolar M, Nicholls J, Ling J. Assessing the feasibility of using health information in alcohol licensing decisions: A case study of seven English local authorities. The Lancet. 2016;388(SPEC.ISS 1):79.	Conference abstract

Moriarty KJ, Gilmore IT. Licensing Britain's alcohol epidemic. JOURNAL OF EPIDEMIOLOGY AND COMMUNITY HEALTH. 2006;60(2):94-.	Editorial
Nicholls J. Alcohol licensing in Scotland: a historical overview. Addiction. 2012;107(8):1397-403.	Description of process or policy (legal and policy framework)
Nicholls J. Public Health and Alcohol Licensing in the UK: Challenges, Opportunities, and Implications for Policy and Practice. Contemporary Drug Problems. 2015;42(2):87-105.	Lacks detail on methods
Reynolds J, Engen J, McGrath M, Pashmi G, Andrews M, Egan M, et al. Influences on public health contributions to alcohol licensing processes in local government in England: A mixed-methods study. The Lancet. 2017;390(SPEC.ISS 1):S77.	Conference abstract
Reynolds J, McGrath M, Halliday E, Ogden M, Hare S, Smolar M, et al. 'The opportunity to have their say'? Identifying mechanisms of community engagement in local alcohol decision-making. The International Journal on Drug Policy. 2020;85:1.	Not focused on public health involvement
Sharpe CA, Poots AJ, Watt H, Franklin D, Pinder RJ. Controlling alcohol availability through local policy: an observational study to evaluate Cumulative Impact Zones in a London borough. JOURNAL OF PUBLIC HEALTH. 2018;40(3):E260-E8.	Quantitative
Thom B, Herring R, Bayley M, Waller S, Berridge V. Partnerships: survey respondents' perceptions of inter-professional collaboration to address alcohol-related harms in England. Critical Public Health. 2013;23(1):62-76.	Not focused on licensing decisions
Ure C, Burns EJ, Hargreaves SC, Hidajat M, Coffey M, de Vocht F, et al. How can communities influence alcohol licensing at a local level? Licensing officers' perspectives of the barriers and facilitators to sustaining engagement in a volunteer-led alcohol harm reduction approach. The International Journal on Drug Policy. 2021;98:1.	Not focused on public health involvement
Wright A. Local Alcohol Policy Implementation in Scotland: Understanding the Role of Accountability within Licensing. INTERNATIONAL JOURNAL OF ENVIRONMENTAL RESEARCH AND PUBLIC HEALTH. 2019;16(11).	Not focused on public health involvement

Citation searching

Report of study	Reason for exclusion
Jankhotkaew J, Casswell S, Huckle T, Chaiyasong S, Phonsuk P. Barriers and Facilitators to the Implementation of Effective Alcohol Control Policies: A Scoping Review. Int J Environ Res Public Health. 2022 May 31;19(11):6742.	Not focused on licensing decisions
R. O’Donnell, A. Mohan, R. Purves, N. Maani, C. Angus, M. Egan & N. Fitzgerald (2023) Mechanisms of impact of alcohol availability interventions from the perspective of 63 diverse alcohol licensing stakeholders: a qualitative interview study, Drugs: Education, Prevention and Policy	Description of policy or process (policy mechanism)

Website searching for grey literature

Organisation	Report of study	Reason for exclusion
Alcohol Change UK	Using licensing to protect public health: from evidence to practice	Lacking detail on methods
	Evaluation of the impact of public health transfer to local authorities on alcohol	Not focused on licensing decisions
Alcohol Focus Scotland	Changing Scotland's relationship with alcohol	Recommendations for government
	Community toolkit	Guidance document
	Licensing resource pack	Guidance document
	Taking Stock: Views and experiences of alcohol licensing in Scotland	Lacking detail on methods
	Glasgow Regional Licensing Event 2016 - event summary	Lacking detail on methods
	Aberdeen Regional Licensing Event 2016 - event summary	Lacking detail on methods
	Dundee Regional Licensing Event 2016 - event summary	Lacking detail on methods
	Edinburgh Regional Licensing Event 2016 - event summary	Lacking detail on methods
Alcohol Health Alliance	Health First: An evidence-based alcohol strategy for the UK.	Recommendations for government
ADPH (Association of the Directors of Public Health)	Policy position: alcohol	Not focused on licensing decisions
	ADPH Consultation Response: Licensing Act 2003 Regulatory Easements	Not focused on public health involvement
Alcohol Licensing Community	Alcohol licensing toolkit	Guidance document
GOV.uk	Alcohol licensing: a guide for public health teams	Guidance document
	A practical approach to making representations to a licensing authority	Guidance document
	Findings from the pilot of the analytical support package for alcohol licensing	Description of process or policy (process and use of analytical tool)
	Alcohol licensing: evaluating public health evidence in Medway	Description of process or policy (process)
	Collaborating on Newcastle's Statement of Licensing Policy review	Description of process or policy (process)
	Considering public health for Liverpool Kensington and Fairfield licence applications	Description of process or policy (process)
	Embedding public health in Coventry's licensing policy statement	Description of process or policy (process)
	Reducing alcohol related health harms in Leeds	Description of process or policy (process)
	Using health data to inform alcohol licensing decisions in Solihull	Description of process or policy (process)
Institute of Alcohol Studies	Anytime, anyplace, anywhere? Addressing physical availability of alcohol in Australia and the UK	Lacking detail on methods

	The Licensing Act (2003): its uses and abuses 10 years on	Not focused on licensing decisions
	Licensing in practice: the availability of alcohol in UK society	Description of process or policy
	Licensing Law	Description of process or policy (policy and legal framework)
Local Government Association	LGA Survey: Public Health and the Licensing Process	Lacking detail on methods
	Public health and alcohol licensing in England	Guidance document
	Public health and the Licensing Act 2003	Guidance document
NIHR School for Public Health Research	Final report: Identifying mechanisms to engage the community in local alcohol decision making. Insights from the CELAD study	Not focused on public health involvement
Public Health Wales	A Guide to Public Health and Alcohol Licensing	Guidance document
Scottish Health Action on Alcohol Problems (SHAAP)	Re-thinking alcohol licensing	Not focused on public health involvement
	Licensing for Public Health	Guidance document

Key author searching

**Report of study**	**Reason for exclusion**
Djordjevic F, Wilkinson C, Reynolds J, Dwyer R, Hickman P. Public health and public participation: a critical comparison of liquor licensing legislation in Australia and the United Kingdom. DRUG AND ALCOHOL REVIEW. 2021;40:S67-S.	Conference abstract
Fitzgerald N, Angus K, Elders A, de Andrade M, Raistrick D, Heather N, et al. Weak evidence on nalmefene creates dilemmas for clinicians and poses questions for regulators and researchers. ADDICTION. 2016;111(8):1477-87.	Not focused on licensing decisions
Fitzgerald N, Lewsey J. Commentary on Wicki et al. (2020): Strong effectiveness evidence-but what else do policymakers need? ADDICTION. 2020;115(8):1470-1.	Not focused on licensing decisions
Fitzgerald N, Uny I, Brown A, Eadie D, Ford A, Lewsey J, et al. Managing COVID-19 Transmission Risks in Bars: An Interview and Observation Study. JOURNAL OF STUDIES ON ALCOHOL AND DRUGS. 2021;82(1):42-54.	Not focused on licensing decisions
Haghpanahan H, Lewsey J, Mackay DF, McIntosh E, Pell J, Jones A, et al. An evaluation of the effects of lowering blood alcohol concentration limits for drivers on the rates of road traffic accidents and alcohol consumption: a natural experiment. LANCET. 2019;393(10169):321-9.	Not focused on licensing decisions
Nicholls J. UK news reporting of alcohol: An analysis of television and newspaper coverage. DRUGS-EDUCATION PREVENTION AND POLICY. 2011;18(3):200-6.	Not focused on licensing decisions
Nicholls J. Time for reform? Alcohol policy and cultural change in England since 2000. BRITISH POLITICS. 2012;7(3):250-71.	Not focused on licensing decisions

O'Donnell R, Mohan A, Purves R, Maani N, Egan M, Fitzgerald N, et al. Navigating different public health roles in alcohol premises licensing: a multi-stakeholder interview study. LANCET. 2021;398:14-.	Conference abstract
Reynolds J, Wilkinson C. Accessibility of 'essential' alcohol in the time of COVID-19: Casting light on the blind spots of licensing? DRUG AND ALCOHOL REVIEW. 2020;39(4):305-8.	Description of process or policy (policy framework and scope)

Reference list review

**Report of study**	**Reason for exclusion**
Fitzgerald, N., Manca, F., Uny, I., Martin, J.G., O'Donnell, R., Ford, A., Begley, A., Stead, M. and Lewsey, J. (2022), Lockdown and licensed premises: COVID-19 lessons for alcohol policy. Drug Alcohol Rev., 41: 533-545.	Not focused on public health involvement
Iconic Consulting. (2014). Strengthening the community voice in alcohol licensing decisions in Glasgow. Final report	Not focused on public health involvement
Jenkins LM, Bramwell D, Coleman A, Gadsby EW, Peckham S, Perkins N, Segar J. Integration, influence and change in public health: findings from a survey of Directors of Public Health in England. J Public Health (Oxf). 2016 Sep;38(3):e201-e208.	Not focused on licensing decisions
Mooney JD, Sattar Z, de Vocht F & Ling J (2022) Assessing the feasibility of using place-based health information in alcohol licensing: case studies from seven local authorities in England, Cities & Health, 6:3, 575-586.	Description of process or policy (process and use of analytical tool)
Select Committee on the Licensing Act 2003. (2017). The Licensing Act 2003: post-legislative scrutiny - HL Paper 146.	Not focused on public health involvement
Toner, P., Lloyd, C., Thom, B., MacGregor, S., Godfrey, C., Herring, R., & Tchilingirian, J. (2014). Perceptions on the role of evidence: an English alcohol policy case study. *Evidence & Policy*, *10*(1), 93-112.	Not focused on licensing decisions

5. Quality appraisal results

CASP qualitative checklist				
Section A: Are the results valid?	**1. Qualitative interviews with public health stakeholders**	**2. PHAL study**	**3. Public health involvement in alcohol licensing**	**4. ExILEnS**
**Was there a clear statement of the aims of the research?**	Yes<br>Identify how individuals working in PH and their organisations have attempted to influence local alcohol licensing policies and decisions.<br>Identify, in their experience, what factors are helpful and/or hindering their efforts to influence.<br>*Secondary aims (2a):*<br>Explore PH stakeholders' perceptions of the distribution of power within the licensing process and how such power is manifested and maintained<br>Analyse current and potential mechanisms of influence in the licensing process as experienced by PH actors.	Yes<br>To explore the range of influences on PHTs contributions to alcohol licensing processes.<br>To identify ways to strengthen PH contributions to alcohol licensing processes.<br>To examine and evaluate the use of a toolkit to support PH practitioners to engage with licensing decisions.	Yes<br>To explore how national policy around the role of PH in alcohol licensing is translated and implemented at a LA level.<br>To identify factors that facilitate or impede PH engagement in alcohol licensing partnerships	Yes<br>*Overall aims of the study were:*<br>To explore how levels of engagement, acceptability, processes and outcomes vary between Scotland and England, and between different PHTs.<br>*Those specific to the qualitative element of the study were:*<br>To explore and understand PH stakeholders' approaches and aims in engaging with alcohol licensing, including their rationale<br>To explore and understand the views of licensing stakeholders on the acceptability and value of these approaches.

**Is a qualitative methodology appropriate?**	Yes<br><br>Qualitative methodology appropriate for both the primary and secondary aims.	Yes<br><br>Appropriate for all aims, although some also required quantitative analysis.	Yes<br><br>Appropriate for both aims and fully justified.	Yes<br><br>Mixed methods study, but qualitative methodology appropriate for stated aims.
**Was the research design appropriate to the aims of the research?**	Yes<br><br>Research design appropriate and briefly justified (semi-structured interviews).	Yes<br><br>Mixed methods, with a mix of qualitative methods used. These were appropriate for the research aims.	Yes<br><br>Combination of qualitative methods used to fully address research aims. Methods justified by author.	Yes<br><br>Research design appropriate to the aims although not explicitly justified.
**Was the recruitment strategy appropriate to the aims of the research?**	Yes<br><br>Recruitment strategy described and discussion of justification of sample size and composition. Focused only on Scotland, which was implicit in the aims, and only included actors who had recent and in-depth experience of engagement in licensing activity.	Yes<br><br>Focused on LAs in London. Aims were not specific to London LAs. Likely the results have external validity for other LAs in England, although likely some bias towards those more engaged in licensing activity.	Yes<br><br>Thorough explanation of recruitment. Focused on LAs in London. Aims were not specific to London LAs. Likely the results have external validity for other LAs in England. Attempts made to recruit a mix of high, medium and low engagement LAs.	Yes<br><br>Detailed description and justification of recruitment strategy. Acknowledgement limitation that PHTs with limited or no engagement in alcohol licensing were not included in the qualitative part of the study.
**Was the data collected in a way that addressed the research issue?**	Yes<br><br>Data collection fully described. Topic guide provided.	Yes<br><br>Clear overview and justification of data collection. Full details of individual methods provided including interview topic guide.	Yes<br><br>Detailed description of data collection and development of methods. Interview questions provided.	Yes<br><br>Data collection methods described in detail. Interview topic guides provided.

**Has the relationship between researcher and participants been adequately considered?**	No<br><br>No reflexive discussion, although it is declared that six participants were known (four well-known) to the interviewer prior to the interview.<br><br>It is also acknowledged that a key informant who was a contact of the PI provided advice on potential participants. Their association was with this informant and the organisation they represent was critically reflected on.	No<br><br>No explicit reflection, however, the researchers do describe how practitioners were involved in shaping the study design, and they reflect on the implications of the recruitment sample and a change in scope to the original research aims (not feasible to address aim 2).	Yes<br><br>Detailed reflexive account provided, reflecting on the potential impact of the researcher being an insider.	No<br><br>No detail of reflections provided in study outputs. However, competing interests are declared, and noted that potential biases within the research team were addressed through reflexive discussion.
Section B: What are the results?	**1. Qualitative interviews with public health stakeholders**	**2. PHAL study**	**3. Public health involvement in alcohol licensing**	**4. ExILEnS**
**Have ethical issues been taken into consideration?**	Yes<br><br>Ethical approval and considerations, and consent process, described.	Yes<br><br>Description of ethical approval and consent for each method provided.	Yes<br><br>Detailed discussion of how ethical considerations were addressed. Ethical approval and consent described.	Yes<br><br>Ethical approval and consent process described.
**Was the data analysis sufficiently rigorous?**	Yes<br><br>Methods described, coding framework used in analysis provided. Data presented supports findings No reflexive account provided.	Yes<br><br>Thorough description of analysis and sufficient data provided to support findings. No reflexive account provided.	Yes<br><br>In-depth discussion of data analysis process. Data presented supports findings. Detailed reflexive account.	Yes<br><br>Clear description of data analysis methods. Data provided to justify findings. No reflexive account provided.

**Is there a clear statement of findings?**	Yes<br>Findings clearly described and discussed in relation to aims, and linked to existing theory where relevant.	Yes<br>Clear discussion of findings, triangulated with existing knowledge and linked to original study aims.	Yes<br>Findings clearly presented and linked to original research aims.	Yes<br>Findings clearly provided and discussed in relation to existing research and research aims.
Section C: Will the results help locally?	**1. Qualitative interviews with public health stakeholders**	**2. PHAL study**	**3. Public health involvement in alcohol licensing**	**4. ExILEnS**
**How valuable is the research?**	Suggestions for further research and learning for PHTs provided. Discussed in relation to existing theory on policy-implementation and power distribution.	Discussion of what the study adds is provided, both in terms of improved understanding and through recommendations to strengthen PHTs' contributions.	Study draws five main conclusions with implications for practice and suggests areas for future research.	Several areas for future research are suggested. Contribution of the study to existing understanding discussed.

6. CERQual Qualitative Evidence Profile

Summary of review finding	Reports of studies contributing to the review finding	Methodological limitations	Coherence	Adequacy	Relevance	CERQual assessment of confidence in the evidence	Explanation of CERQual assessment
**1. The PHT role: variation in interpretation and approaches**							
There are different interpretations of the role of PHTs and the way teams enact this through the work they do.	1a,b<br>2a,c<br>3a,b<br>4a	Minor concerns regarding methodological limitations. Three studies lacked adequate representation of PHTs with limited or no engagement in licensing work.	No or very minor concerns regarding coherence.	No or very minor concerns regarding adequacy.	No or very minor concerns regarding relevance.	High confidence	Three studies with minor concerns regarding methodological limitations. No or very minor concerns across the four domains.
PHTs demonstrate differing approaches to engagement with other licensing stakeholders.	1b<br>2a<br>3a,b<br>4a	Minor concerns regarding methodological limitations. Three studies lacked adequate representation of PHTs with limited or no engagement in licensing work.	No or very minor concerns regarding coherence.	No or very minor concerns regarding adequacy.	No or very minor concerns regarding relevance.	High confidence	Three studies with minor concerns regarding methodological limitations. No or very minor concerns across the four domains.

**2. Beyond individual licensing decisions: strategic involvement**							
Some PHTs prioritise building relationships and raising the public health profile over trying to influence individual licensing decisions.	1b<br>2a,c<br>3a<br>4a,b	Minor concerns regarding methodological limitations. Three studies lacked adequate representation of PHTs with limited or no engagement in licensing work.	Minor concerns regarding coherence. Some variation in how explicit studies were regarding raising PH profile.	No or very minor concerns regarding adequacy.	No or very minor concerns regarding relevance.	High confidence	Four studies with minor concerns regarding methodological limitations and coherence, but no or very minor concerns in the other domains.
Some PHTs prioritise involvement in upstream, population-based work such as SLPs and CIPs, rather than individual licensing decisions.	2a,b,c<br>4a,b	Minor concerns regarding methodological limitations. Two studies lacked adequate representation of PHTs with limited or no engagement in licensing work.	No or very minor concerns regarding coherence.	Minor concerns regarding adequacy. Two studies do not refer to this, but this does not refute the finding. Rich data in the others.	No or very minor concerns regarding relevance.	High confidence	Two studies with minor concerns regarding methodological limitations and adequacy, but no or very minor concerns in the other domains.
**3. Last among equals?**							
PHTs have faced barriers as a result of coming in as an outsider to licensing, which has pre-established processes and actors.	1a,c<br>3a,b	Minor concerns regarding methodological limitations. One study lacked adequate representation of PHTs with limited or no	No or very minor concerns regarding coherence.	Moderate concerns regarding adequacy. Two of the four studies do not raise this. Of the two that do, the data is very rich in one, but less so in the other.	No or very minor concerns regarding relevance.	Moderate confidence	Two studies, with moderate concerns regarding adequacy and minor concerns regarding methodological limitations. No or very minor concerns in the other domains.

		engagement in licensing work.					
PHTs are perceived as having an unequal status and a more supporting role compared to other licensing stakeholders.	1a,c<br>2a,b<br>3a,b<br>4a,b	Minor concerns regarding methodological limitations. Three studies lacked adequate representation of PHTs with limited or no engagement in licensing work.	Minor concerns regarding coherence. Slight differences in the way the supporting role was described.	No or very minor concerns regarding adequacy.	No or very minor concerns regarding relevance.	High confidence	Four studies with minor concerns regarding methodological limitations and coherence, but no or very minor concerns in the other domains.
The public health licensing objective is perceived, in Scotland, to be a lesser priority; in England, its absence means public health considerations appear less valued.	1b,c<br>2a<br>3a,b<br>4a,4b	Minor concerns regarding methodological limitations due to the studies' settings: two in Scotland, one in England, one covering both. Three studies lacked adequate representation of PHTs with limited or no engagement in licensing work.	Minor concerns regarding coherence. There were nuances in how this finding was expressed.	No or very minor concerns regarding adequacy.	No or very minor concerns regarding relevance.	High confidence	Four studies with minor concerns regarding methodological limitations and coherence. No or very minor concerns in the other domains.

**4. Professional cultures**							
There are differences in approaches between PHTs and other licensing stakeholders as to how evidence is defined and what is considered valuable and relevant.	1b,c<br>2a,b<br>3a,b<br>4a	No or very minor concerns regarding methodological limitations.	No or very minor concerns regarding coherence.	No or very minor concerns regarding adequacy.	No or very minor concerns regarding relevance.	High confidence	Four studies with no or very minor concerns across the four domains.
There are differences in beliefs about alcohol and the aims of licensing between PHTs and other licensing stakeholders.	1b,c<br>2a<br>3a<br>4a	No or very minor concerns regarding methodological limitations.	Minor concerns regarding coherence. Emphasis varied between studies.	No or very minor concerns regarding adequacy.	No or very minor concerns regarding relevance.	High confidence	Four studies with minor concerns regarding coherence. No or very minor concerns across the other domains.
The licensing process is a form of quasi-judicial decision-making, and is unfamiliar to PHTs.	1a,c<br>2a<br>3a	Minor concerns regarding methodological limitations. Two studies lacked adequate representation of PHTs with limited or no engagement in licensing work.	No or very minor concerns regarding coherence.	Minor concerns regarding adequacy. This was not raised in one of the studies.	No or very minor concerns regarding relevance.	High confidence	Three studies, with minor concerns regarding coherence, and minor concerns regarding methodological limitations in two. No or very minor concerns in the other domains.
The prioritisation of licensing work and the capacity within PHTs to undertake it.	1c<br>2a,b,c	Moderate concerns regarding methodological limitations. Both lacked adequate representation of PHTs with limited or no	No or very minor concerns regarding coherence.	Moderate concerns regarding adequacy. Not raised by two of the four studies, but rich data in the two that did raise it.	No or very minor concerns regarding relevance.	Moderate confidence	Two studies with moderate concerns regarding methodological limitations and adequacy. No or very minor concerns in the other domains.

		engagement in licensing work.					
**5. A long-term approach**							
Taking the time to build relationships with other licensing stakeholders is seen as beneficial for PHTs.	1b,c 2a,b 3a,b 4a,b	No or very minor concerns regarding methodological limitations.	No or very minor concerns regarding coherence.	No or very minor concerns regarding adequacy.	No or very minor concerns regarding relevance.	High confidence	Four studies with no or very minor concerns across the four domains.
It is beneficial for PHTs to plan for licensing work to require sustained involvement.	1c 2a 3a,b	No or very minor concerns regarding methodological limitations.	Moderate concerns regarding coherence. This was expressed in different ways, with varying specific proposals.	Moderate concerns regarding adequacy. Not raised by one study and richness of data was generally limited.	No or very minor concerns regarding relevance.	Moderate confidence	Three studies with moderate concerns regarding coherence and adequacy. No or very minor concerns regarding relevance or methodological limitations.
It is beneficial for PHTs to increase their relevant knowledge and skills, such as understanding of the licensing process and communication of evidence.	1c 2a,b 3a,b	No or very minor concerns regarding methodological limitations.	No or very minor concerns regarding coherence.	Minor concerns regarding adequacy. Richness of the data varied and was not raised by one study.	No or very minor concerns regarding relevance.	High confidence	Three studies with minor concerns regarding adequacy. No or very minor concerns in the other three domains.
**6. A public health licensing objective: necessary but not sufficient?**							
A public health licensing objective is seen as beneficial, if not necessary, in theory in England and in practice in Scotland, but it is not seen as sufficient for effective PH involvement.	1a,b,c 2a,b 3a,b 4b	Minor concerns regarding methodological limitations due to the studies' settings: two in Scotland, one in England, one covering both.	Minor concerns regarding coherence. Nuances in how this finding was expressed, within and between studies.	No or very minor concerns regarding adequacy.	No or very minor concerns regarding relevance.	High confidence	Four studies with minor concerns regarding methodological limitations and coherence. No or very minor concerns regarding adequacy and relevance.

**7. Contested value of PHT involvement**							
There are variations among PHTs and other licensing stakeholders in the perceived value of PHT involvement in licensing decisions.	1a,b; 2a,b,c; 3a; 4a	Minor concerns regarding methodological limitations. Three studies lacked adequate representation of PHTs with limited or no engagement in licensing work, but they still found variation. One study lacked adequate discussion of researcher reflexivity.	No or very minor concerns regarding coherence.	Minor concerns regarding adequacy. Richness of the data was limited in one of the studies.	No or very minor concerns regarding relevance.	High confidence	Four studies with minor concerns in three regarding methodological limitations and minor concerns in one regarding adequacy, but these concerns do not threaten the finding itself as variation is demonstrated regardless. No or very minor concerns regarding coherence or relevance.
The allocation of PHT resources for involvement in licensing decisions could be questioned in light of PHTs' potentially limited impact.	1b, 2c; 3a; 4a	Moderate concerns regarding methodological limitations. Two studies lacked adequate discussion of researcher reflexivity. Three studies lacked adequate representation of PHTs with limited or no	Moderate concerns regarding coherence. One study does not raise this issue, which could imply a contradiction, though it does find variation in perceived impact. In two studies this is a second-order interpretation.	Moderate concerns regarding adequacy. Richness and quantity of data is limited.	No or very minor concerns regarding relevance.	Moderate confidence	Four studies with moderate concerns regarding coherence, adequacy and methodological limitations. No or very minor concerns regarding relevance.

		engagement in licensing work.	The third study does not frame it as a normative finding, although this is the implication of the participant's quote.				
